# “A welfare recipient may be drinking, but as long as he does as told – he may drink himself to death”: a qualitative analysis of project implementation barriers among Danish job consultants

**DOI:** 10.1186/s12889-015-1620-x

**Published:** 2015-03-18

**Authors:** Maja Bæksgaard Hansen, Stine Kloster, Ida Høgstedt Danquah, Anette Søgaard Nielsen, Ulrik Becker, Tine Tjørnhøj-Thomsen, Janne Schurmann Tolstrup

**Affiliations:** National Institute of Public Health, University of Southern Denmark, Øster Farimagsgade 5A, Copenhagen, DK-1353 Denmark; Unit of Clinical Alcohol Research, Institute of Clinical Research, University of Southern Denmark, JB Winsløwsvej 20, Entrance 220B, 1st floor, Odense, C DK-5000 Denmark

**Keywords:** Implementation, Intervention, Process evaluation, Randomised controlled trial, Alcohol, Employment

## Abstract

**Background:**

This paper is embedded in a randomised controlled trial (Alcohol and Employment) that investigated whether welfare-to-work schemes combined with alcohol treatment were more effective than welfare-to-work schemes alone for helping unemployed welfare recipients with alcohol problems get back to employment and reduce their alcohol problems. The implementation of Alcohol and Employment turned out to be challenging, and fewer welfare recipients than expected were enrolled. The aim of this paper was to identify and investigate obstacles to the implementation of Alcohol and Employment. Our main objective was to study the job consultants’ role in the implementation process as they were key personnel in conducting the trial.

**Methods:**

The process evaluation was conducted in four Danish municipalities in 2011–2012. Data for identifying factors important for the implementation were collected through observations and focus group interviews with job consultants. Data were analysed thematically and thoroughly discussed among members of the project team; emerging themes were then grouped and read again repeatedly until the themes were consistent.

**Results:**

Three themes emerged as the main factors influencing the degree of implementation of Alcohol and Employment: (1) The job consultants’ personal attitudes toward alcohol were an important factor. The job consultants generally did not consider a high alcohol intake to be an impediment to employment, or they thought that alcohol problems were only symptoms of more profound problems. (2) The job consultants’ perception of their own roles and responsibilities in relation to the welfare recipients was a barrier: they felt that addressing alcohol problems and at the same time sustaining trust with the welfare recipient was difficult. Also, they did not consider alcohol problems to be their responsibility. (3) Shortage of time and resources among the job consultants was determined to be an influential factor.

**Conclusions:**

We identified important factors at the individual level among the job consultants who threatened the implementation of Alcohol and Employment. Future studies in similar settings can take advantage of these findings when preparing interventions that are implemented by job consultants or similar professionals.

**Trial registration:**

ClinicalTrials.gov ID: NCT01416103.

## Background

In 2007, Danish municipalities became responsible for executing disease prevention and health promotion as a consequence of structural reform [1]. Examples include prevention of excessive alcohol use and alcohol treatment. Recent research has shown that the municipalities have no systematic approach toward alcohol problems among unemployed welfare recipients, despite the fact that 33% of this population has an alcohol problem to such an extent that it constitutes a barrier to employment [2-4]. Hence, there is an obvious need for an effective preventive approach.

A systematic review of reviews of controlled trials has shown that screening for alcohol problems by questionnaire followed by brief intervention significantly reduces alcohol consumption in primary care populations [5]. Efficacy has also been demonstrated in health screening programmes in general patients [6-8] and student settings [9]. Evidence from emergency departments and hospital settings is mixed [10] or inconclusive [11]. In principle, job consultants have unique opportunities to identify alcohol problems and to refer to treatment, because they have regular face-to-face contact with the welfare recipients. It is, however, unknown whether a focussed effort on alcohol problems can help welfare recipients reduce their alcohol consumption and return to work. Danish studies targeting heavy drinkers in settings outside primary health care have not been performed.

We conducted a randomised controlled trial (Alcohol and Employment) among unemployed welfare recipients with alcohol problems in four Danish municipalities in 2011–2012. The purpose was to use the municipalities’ already existing programmes for employment (welfare-to-work schemes) and alcohol treatment. The job consultants’ task was to identify welfare recipients with alcohol problems by systematic screening. Those who agreed to participate in the study were subsequently randomised to either an intervention group receiving the usual welfare-to-work scheme combined with alcohol treatment or a control group receiving the usual welfare-to-work scheme only. After 6 and 12 months, welfare recipients were contacted by telephone and asked about their alcohol use and attachment to the labour market.

This rather simple intervention turned out to be difficult to implement. Only a third of the anticipated number of welfare recipients was enrolled. In order to elucidate the causes of this difficulty, qualitative interviews were conducted with the job consultants. The aim of this paper was to identify and investigate obstacles to the implementation of Alcohol and Employment. Our main objective was to study the job consultants’ role in the implementation process as they were key personnel in conducting the trial.

### Theoretical perspective

Our theoretical framework is influenced by Winter’s Integrated Implementation Model [12] and Lipsky’s concept of street-level bureaucrats [13]. The theoretical perspective should be seen only as a framework presenting key factors and mechanisms that may affect implementation. We used the literature to specify factors that could be important for the implementation of Alcohol and Employment and to analyse the results. The framework was also used for developing the interview guide.

#### Winter’s integrated implementation model

Winter’s Integrated Implementation Model suggests three sets of factors that affect the implementation process. The first set concerns the process of policy formulation and design [14]. The structural reform that was introduced in 2007, making Danish municipalities responsible for executing disease prevention and health promotion, can be perceived as a new policy [1]. This policy was, however, not specifically defined and leaves the Danish municipalities with a great degree of freedom, also due to the municipalities’ right to self-government [15]. The first set of factors in Winter’s Integrated Implementation Model can be used to understand the setup and context that the new health policy created in the Danish municipalities. The intervention project Alcohol and Employment is a direct result of the new health policy. The four participating municipalities implemented the project in an attempt to execute disease prevention and health promotion.

The second set of factors focusses on three levels of the implementation process: (1) organisational and interorganisational behaviours, representing the degree of commitment, coordination arrangement, readiness, innovation, and leadership [16-21]; (2) individual factors affecting the behaviours of street-level bureaucrats, including knowledge, norms, needs, and beliefs, as well as the perceived benefits and disadvantages of the interventions [16,22,23]; and (3) the behaviours of the target group of public policies. In the results section we will demonstrate that these factors are relevant for the analysis of the obstacles in the project Alcohol and Employment, especially factors related to street-level bureaucrats, in this case the job consultants.

The third set of factors, such as external pressures and ups and downs in the business cycle, concern the socioeconomic context. The municipalities have experienced major budget restrictions. Since 2009 the municipalities have reduced service costs by more than £12 billion, resulting in adjustments in a number of service areas, such as employment administration [24]. Such factors also affected this project both in the recruitment of the municipalities and the job consult in the implementation process, which is described in more detail in the results section.

#### Lipsky’s street-level bureaucrats

The behaviours of street-level bureaucrats are crucial for implementation of most policies [12], and Lipsky’s (1980) insights into street-level bureaucrats are also part of Winter’s Integrated Implementation Model [12].

According to Lipsky, the actions of street-level bureaucrats often differ from what is intended at the political level, such as in the case of implementing a new standard [13]. A number of studies have attempted to identify the extent and cause of this divergence. Street-level bureaucrats are in direct contact with the welfare recipients and thus provide the service delivered by local government [13]. Their work is characterised by many demands and limited resources, and they cope with this situation by modifying policy design and formulation, and thereby rationing services and making priorities among projects and welfare recipients [23].

The project Alcohol and Employment can be perceived as a new standard that widens the job consultants’ focus to also include potential alcohol problems. Standards, along with norms and directives, express a code of practice informing actors what to do in specific situations [25]. Standards differ from norms in being more specific and having an obvious source. At the same time, standards differ from directives in the sense that standards are not obligatory; rather, organisations can voluntarily adopt them or not [26,27]. A standard can have various characteristics which render it more or less prone to diffuse: that is, some standards will diffuse quickly whereas others will spread slowly or not at all [26]. In implementing Alcohol and Employment, job consultants act as gatekeepers, linking the system and the welfare recipient. To understand the implementation process and diffusion of Alcohol and Employment, it is important to understand the factors that may influence the job consultants’ work in this regard.

### Alcohol and employment – setting and design of the intervention

In Denmark, welfare recipients receive a monthly social benefit from the municipality or – if the recipient is insured – from an unemployment fund. The recipients must be deemed available for work in order to qualify for benefits. All welfare recipients are categorised into three groups based on qualifications and personal resources. Welfare recipients who are prepared to attend an ordinary job are categorised as Match 1; welfare recipients who are not prepared immediately but at the same time are qualified for job preparation activities, as Match 2; and those who are not ready for job preparation activities, as Match 3 [28].

In the municipalities, job consultants work directly with welfare recipients. Job consultants are mostly social workers by education, but other professions can also fill this function. Major tasks include categorising recipients into Match groups, arranging and coordinating services, giving advice on job searches and career guidance, and placing welfare recipients into employment-promoting activities (welfare-to-work schemes). Common to all initiatives is a focus on guiding welfare recipients back to the labour market. Finally, job consultants have to follow a number of directives and verify that welfare recipients are deemed available for work [29].

### The intervention

Alcohol and Employment was designed as a randomised controlled trial. Welfare recipients from all three Match groups were included. Initially, job consultants screened all the welfare recipients for problematic alcohol use with the Alcohol Use Disorder Test (AUDIT), which is a screening tool developed by the World Health Organization for clinical use to identify people with hazardous or harmful levels of alcohol consumption, as well as alcohol dependence [30,31]. Recipients with an AUDIT score of 8 or above were eligible for enrolment [32,33] and were randomised to follow the usual welfare-to-work scheme or the usual welfare-to-work scheme combined with alcohol treatment.

#### Welfare-to-work schemes

Welfare recipients in both the control and intervention group were referred to the welfare-to-work schemes deemed most appropriate by the job consultants. Welfare-to-work schemes represent the ‘standard procedure’ including, for example, job search courses, help with CV and applications or establish practices and subsidized jobs.

#### Alcohol treatment

Welfare recipients in the intervention group were referred to outpatient alcohol treatment programmes that already existed in the four municipalities. Treatment programmes are tailored to individual needs and characteristics and include, for example, cognitive therapy, supportive conversations, family therapy, and medical care.

In three of the four municipalities (Site A, B, and D), the job consultants performed the screening as part of a routine meeting with welfare recipients and afterward referred the welfare recipient to a colleague. The colleague then offered the welfare recipients enrolment in the project and assisted them with the completion of a questionnaire about their employment status, alcohol consumption, other habits of abuse, health status, and background information. In a fourth municipality, the responsibility for enrolment was allocated entirely to the job consultants, and they therefore performed both the screening and the enrolment procedure.

The design of Alcohol and Employment was pilot tested in one municipality, and the specific design was adapted as a consequence. In 2011–2012, four municipalities were included in the study. The decision to participate was made at the management level at the job centres in the municipalities. We learned that each municipality – not to mention the various branches in each municipality – had its own way of organising, administering, and managing its work. The principal design of Alcohol and Employment was similar at all four sites, but in order to embrace the differences in each municipality, it was carried out with minor variations. Specific procedures were planned with the job consultants who were to carry out the intervention. The involvement of the job consultants varied locally. In three municipalities (Sites B, C, and D), job consultants received relevant training from clinically experienced alcohol researchers prior to the implementation of the project. The management at Site A stated that the job consultants already had sufficient experience and training, and thus did not wish to participate in the training. A total of 1544 welfare recipients were screened; 359 scored 8 or above on the AUDIT scale, and 113 were enrolled in the intervention project.

## Methods

This paper reports qualitative results from the project Alcohol and Employment. Data for identifying factors important for the implementation of the project were collected through focus group interviews supplemented by structured field notes in all four municipalities (Sites A, B, C, and D).

### Participants

All job consultants working with the project were offered the chance to participate in focus group interviews. Due to practicalities, selection differed between municipalities, but in all sites the key persons in the project were included.

### Interviews

During the study period, we conducted structured field notes in each municipality, observed routine practice, and had informal discussions with job consultants at the job centre. This provided insight into the culture and norms at each site. The development of the interview was guided by our program theory, the structured field notes, and prespecified factors hypothesised to be important `for implementation, which were derived from the literature [12-14,16-23]. The interview guide thus contained questions on factors such as procedures in the specific municipality, organisational behaviour, the quality of cooperation between the job centre and the alcohol treatment institution, and the job consultants’ knowledge, norms, needs, beliefs, and perceived benefits and disadvantages of the intervention.

Traditionally, a focus group interview is defined as a method whereby data are produced by interaction and dialogue in a group consisting of two or more individuals around a set of issues chosen by the interviewer beforehand. The focus group interview is particularly suited to shed light on the social significance of phenomena [34,35]. In this study, focus group interviews yielded information about job consultants’ experiences, points of view, and knowledge. Data from the interviews also provided detailed descriptions of interpersonal interactions and organisational processes [35]. The focus group interviews were conducted as semistructured focus group interviews with open-ended questions, allowing themes other than those chosen prior to the interview to emerge [36].

We conducted four focus group interviews. Interviews were conducted during the period from March to September 2012, at the end of the intervention period. Each interview involved two to six job consultants and lasted around one hour. Interviews continued until no new themes arose. The interviews were audiotaped and transcribed in part by two researchers (MBH and SK) shortly afterward [37].

### Analyses

Data analysis took place in two phases to avoid forcing the data in any given direction. In the initial phase SK and MBH read through the material and used open coding [38]. Coded statements were grouped into categories, which again were grouped into themes. As themes began to emerge, the interviews were categorised accordingly and reread. This continued until themes were consistently defined. In the second phase, themes were discussed with coauthors and underwent a number of iterations until three main themes resulted [35]. Quotations in the results section are used to illustrate key themes as raised by the job consultants.

### Ethics

The intervention project (Alcohol and Employment) was approved by the Danish Data Protection Agency and the Research Ethics Committee of the Capital Region. A multilevel process of consent was used. Head management at the job centres consented for the municipalities to be involved in the project. Welfare recipients who participated in the intervention project gave written consent. Informed consent was also provided by job consultants who participated in the focus group interviews. The job consultants understood that the data collected in this study would be used for research. In addition, data is analysed anonymous on both the municipal and individual level.

## Results

This paper highlights a number of key factors that influenced the job consultants’ implementation of Alcohol and Employment. We aim to present results that clarify why many fewer welfare recipients were enrolled in the project than expected.

Results are presented within Winter’s Integrated Implementation Model [39], together with illustrative quotations. Three themes emerged as main factors that were crucial for the degree of implementation: (1) the job consultants’ personal attitudes toward alcohol; (2) the job consultants’ perception of their own roles and responsibilities; and (3) the job consultants’ shortage of time and resources.

### The job consultants’ personal attitudes toward alcohol

During the interviews the job consultants’ personal attitudes toward alcohol appeared as a theme numerous times, although this topic was not explicitly addressed during the interviews. Like the rest of the Danish population, the job consultants had different attitudes about what defines heavy drinking and what the associated health and social consequences are. This may have affected their appreciation of the project and their judgement of the need to refer welfare recipients to alcohol treatment. This is expressed in the following quotations:

“But some of them are indeed able to function in spite of actually being alcoholics. And indeed, they themselves do not consider it a problem either. If they drink 10 beers a day, it’s actually not a problem if you cannot sense anything on them. Why is it then that all of a sudden we think it’s a problem?” (Site C)

“I too [as a job consultant] have thought many times that alcohol is not a problem, and not at all in relation to getting a job.” (Site D)

“Some people need 10 beers to be able function at all. We have to face up to this fact.” (Site C)

Some of the job consultants did not see the need to interfere with the welfare recipients’ personal lives, including their alcohol habits. This seemed to constitute a barrier to implementation since a main presumption behind the intervention was that alleviating alcohol problems would increase the likelihood of a successful job-seeking process.

Job consultants’ personal attitudes toward and beliefs about alcohol are transferred to their work, as the following comment illustrates:

“Well, sure, we ourselves could have scored 8 on the AUDIT scale.” (Site C)

In this quotation, the job consultant refers to the screening procedure for problem drinking. The job consultant is an example of a professional who does not find heavy alcohol drinking to be a problem. Therefore, he or she does not perceive the welfare recipients as being potential subjects for alcohol treatment. The Danish implementation researcher Søren Winter has demonstrated a similar point. In his research, he shows that job consultants’ values and attitudes toward new standards affect how the standard is implemented [40,41].

At all sites, job consultants highlighted that the welfare recipients have more-serious problems than alcohol, especially for Match categories 2 and 3. Therefore, it seemed meaningless for them to address the alcohol problems with their clients. This point was expressed in the following comments:

“It is, of course, a symptom of an underlying problem, so to me it’s not so important … You can put them into treatment as much as you like, but unless you deal with some of all the underlying problems, then it really makes no difference.” (Site B)

“There are a lot of other barriers too, aren’t there? I don’t have anywhere to live, what can I do? Can you help? There are an awful lot of other things that fill their minds.” (Site D)

“Abuse is, of course, to some extent self-medication. It’s treating something that is painful. That’s what’s so terrible about suddenly focussing so much on alcohol abuse and that they shouldn’t drink, because then they bloody well can’t function.” (Site B)

The above quotations indicate some acceptance of excessive alcohol intake. Job consultants estimated that alcohol treatment would not address the real problem among the welfare recipients, and they had little faith in the intervention. This may be one of the main reasons why the job consultants managed to enrol far fewer welfare recipients in the intervention project than expected.

### The job consultants’ perception of their own roles and responsibilities

Another consistent theme addressed in the interviews was the job consultants’ perception of their own roles and obligations in relation to the welfare recipients. The job consultants experienced a dilemma because, on the one hand, they had to establish a relationship of trust and, on the other, they were obliged to verify that the welfare recipients were adhering to rules and regulations, including being deemed available for work. It also became clear from the interviews that the job consultants were attentive to the limits of their field of work. In many cases, they felt that dealing with alcohol problems was not their responsibility. These facets are further examined below.

The job consultants repeatedly pointed out the importance of sustaining trust with the welfare recipient when discussing problem drinking. The welfare recipients could easily worry about not receiving benefits if they admitted to problematic alcohol consumption. This dilemma was mentioned several times during observations and interviews, as expressed in the following quotation:

“The [alcohol] screening is presented to them at the same time as a lot of directives and regulations, legal requirements, and things they must do and live up to. They feel a bit ill at ease. It’s not exactly then that they open up and say, I might have a problem.” (Site C)

At Site C, job consultants found it difficult to discuss alcohol problems with the welfare recipients because they felt it was necessary to maintain a relationship of trust, and because the issue would make the welfare recipients fear sanctions such as the withholding of benefits.

Some job consultants emphasised that they did not feel that alcohol problems among welfare recipients were their responsibility, as clearly illustrated in the following comment:

“A welfare recipient may be drinking, but as long as he does as told – he may drink himself to death.” (Site A)

This job consultant’s main focus was not to help welfare recipients solve their alcohol problems. He or she was primarily concerned with whether the welfare recipients fulfilled their obligations as stated in employment policy and directives: that is, this individual took on the role of authority only. In fact, job consultants at all sites mentioned that if the welfare recipients did what they were supposed to do, the consultants did not care about their clients’ alcohol use. In such cases, the job consultants deliberately ignored the guidelines of the project, which indicates that the job consultants interpreted the guidelines differently.

### The job consultants’ shortage of time and resources

Major barriers in three of the four municipalities appeared to be a shortage of time and resources. This was the main reason given for the low number of included welfare recipients. In the following discussion, we present factors related to this issue.

A significant factor for successful implementation was whether the job consultant was able to prioritise the intervention project in his or her daily workload. This turned out to be a challenging task, as expressed in the following quotations:

“It’s an extra burden. A working task that has been imposed on us: we weren’t asked. We’ve done it because we MUST.” (Site A)

“But we haven’t had less to do because of it. It was an extra thing we had to do on top of the other things, and it’s like that with many extra things. There was some resistance because of this.” (Site D)

The above quotations reflect frustration among the job consultants. The comments referring to time and conditions vary among sites. The same point is also expressed in the following quotation, but in this case the job consultant points out that tasks associated with implementing Alcohol and Employment were not among the items on which the job consultants were evaluated by their managers:

“It’s not being gauged either – but so many other things are. It’s not assigned point values, but we do score negatively if we don’t keep up our other tasks.” (Site D)

Hence, there were no consequences for not following the project guidelines, and lack of time and resources tipped the job consultants’ priority toward performing the usual tasks over tasks associated with the intervention.

In the following comment, a job consultant addresses the problems of time and the timing of the project:

“If the project had been at a different time, perhaps we would have achieved a different result. Much of it is hack work. Much of it is simply very poorly done work – at least in my case because I am pressed for time and just cannot go on like this.” (Site A)

At Site A, the job consultants pointed out the issue about lack of time even before implementing the intervention, and continued to bring it up at each following meeting. This was probably influenced by a recent round of layoffs, which may have caused them to put extra focus on and feel greater scepticism about the issue of time. A previous study found that employees reacted negatively to having influence over only parts of the intervention project – that is, limited influence over the scope of the problem [42].

At Site C, time aspects were only mentioned briefly. Before implementing the intervention, the job consultants had decided that time issues should not be allowed to interfere with implementing the project. However, when asked directly, they agreed that lack of time constituted a barrier, as expressed in the following comment:

“I know we’re not allowed to say this, but I’m saying it anyway – there’s also a time aspect in it. Already, we don’t have much time for them [welfare recipients].” (Site C)

Although job consultants found the project relevant and believed that the intervention could benefit welfare recipients in the long term, the project still became a “must do” assignment.

At Site D, lack of time and resources was initially not a problem. The site allocated extra resources, but during the project period these were taken up by other tasks, and the time and resources aspect became a barrier here as well. Job consultants at Site B never mentioned lack of time or resources as barriers.

## Discussion

Alcohol and Employment is a randomised controlled trial that tested the effect of alcohol treatment among unemployed welfare recipients. Job consultants in the municipalities primarily implemented the study. They saw value in this intervention project and acknowledged the importance of helping welfare recipients back to employment. Nonetheless, we found that many factors known to impede successful implementation were present at the time that Alcohol and Employment was introduced in the four municipalities. The results presented in this paper outline the major barriers to this implementation, which resulted in far fewer welfare recipients than anticipated. We identified three consistent themes that influenced the implementation. These findings are discussed below.

First, we found that the job consultants’ personal attitudes toward alcohol played a role in the implementation of Alcohol and Employment. They did not consider a high alcohol intake to be a significant impediment to the employment of welfare recipients, or they thought that alcohol problems were only symptoms of other and more profound problems. Danish alcohol culture is liberal. Alcohol is a major part of social life, but at the same time, a high intake is associated with adverse consequences on multiple levels – somatic, psychiatric, and social [43-45]. The Danish population is positioned in the top of the world record in alcohol consumption [45,46] and is also characterised by a very low prevalence of abstainers. Like the rest of the Danish population, each job consultant had different attitudes about how heavy drinking is defined, what its general consequences are, and what its particular impact might be both socially and in terms of job-seeking activities. This might have affected some of the job consultants’ commitment to the Alcohol and Employment project and their perception of the need for the welfare recipients to be involved in alcohol treatment. The job consultants’ behaviour conflicts with the Danish municipalities’ responsibility to execute disease prevention and health promotion [15]. This is consistent with the barrier suggested in Winter’s Integrated Implementation Model [12].

Second, we found that the job consultants’ perception of their own roles and responsibilities in relation to the welfare recipients was a barrier to successful implementation. The job consultants felt to some extent that addressing alcohol problems and at the same time sustaining trust with the welfare recipients was difficult. Also, they did not consider alcohol problems among the welfare recipients to be their responsibility. This set of factors indicates that job consultants are influenced by their professional knowledge, needs, and beliefs, as well as by the perceived benefits and disadvantages of the interventions. These findings are consistent with much of the research on the implementation behaviour of street-level bureaucrats [12,13,16,22,23] as well as research done in other settings [47-49].

The findings in this paper suggest that job consultants deliberately ignored the guidelines of the project. In order to get some understanding of the problem, we turn to the philosopher Charles Taylor (1995). Taylor points out that in real-life settings with human interaction, there will always be endless obstacles or possibilities that arise in the course of any implementation process, in spite of specific guidelines and instructions [50]. Implementing Alcohol and Employment was not mandatory for the job consultants, and there was no control over how they chose to deliver the intervention. According to Lipsky, street-level bureaucrats have a tendency to follow directives in situations where sanctions for not doing so are significant, and a tendency not to follow them where sanctions are trivial [13]. As illustrated, some of the job consultants did not find that alcohol constituted an important problem for the welfare recipients, or they did not think that alcohol problems were their responsibility. Søren Winter has demonstrated a similar point in the Danish context. He shows that how job consultants conduct their job is characterised by whether they perceive a law or regulation as meaningful or not [40,41].

Third, we found that the shortage of time and resources among the job consultants threatened the implementation of Alcohol and Employment. Overall, the job consultants deal with the stress and complexity of their everyday work by themselves, so they made decisions to implement Alcohol and Employment in a way that was possible given their workload. Lipsky (1980) argues that street-level bureaucrats cope with the shortage of time and resources by rationing services, making priorities among projects (such as Alcohol and Employment), and modifying policy goals. According to Lipsky, the coping behaviours of street-level bureaucrats systematically bias the delivery behaviours intended by policy makers [13], or in this case, the overall goal of the project. In this programme, each job consultant decided whether to continue his or her work as before or to adopt the new standard. During the intervention period, job consultants also faced rising caseloads due to a recent round of layoffs. This caused job consultants to focus even more on the lack of time and resources – a key factor also illustrated in Winter’s Integrated Implementation Model [12]. A number of studies suggest that institutional resources and incentives establish the boundaries within which job consultants can act [13,51,52]. Thus, priority given to the implementation of Alcohol and Employment was influenced by other than individual factors, such as management-level failure to supply the job consultants with extra resources for the project. In others words, organisational and interorganisational factors affect the degree of commitment and coordination. A similar point is demonstrated in Johnson’s (2011) systematic review [48] and in Winter’s Integrated Implementation model [12].

In contrast to the robust and comprehensive literature supporting effectiveness in primary health care, the evidence base for alcohol screening and brief intervention in social services, such as job centres, is essentially nonexistent. Although some reviews [49,53] and trials [54-60] have been conducted in such settings, results are inconclusive. The research is far more complex, and there is not a well-founded perception of what works best, in which context, and with whom. A systematic review of qualitative evidence in primary health care settings has identified a number of barriers and facilitators to implementation. Implementation was reported to be limited by lack of resources, training, and support from management, as well as by workload [48]. A similar pattern was discernible in the intervention project Alcohol and Employment.

The Nilsen (2006) systematic review [61] investigated the effectiveness of different strategies to implement brief interventions in primary health care. The systematic review found intervention effectiveness increased with the amount of training by targeting health care providers and/or support from management. Nevertheless, the association was not always straightforward. Studies found that even after training, some health care providers do not carry out an intervention according to protocol [62,63] and some remain unmotivated [64]. Similarly, the Anderson (2004) study [65] observed that the number of alcohol screening and brief interventions increased, but only among health care providers who already felt secure and committed. Training and support did not improve attitudes toward working with drinkers; additionally, it worsened the attitudes of those who were already uncommitted and insecure [65]. In our project three out of four municipalities received training prior to implementation. The one municipality that did not receive training was slightly more distanced and negative toward the project. This may also be due to other organisational barriers, such as the recent round of layoffs and/or lack of support from management. More-substantial initiatives can be assumed to achieve a successful implementation. For instance, the government-supported Risky Drinking project in Sweden has shown positive results by introducing a long-term training and education program while supporting a more prominent place in routine practice [66].

One systematic review, conducted as part of the European Union–financed BISTAIRS research project, aimed to compare barriers and facilitators to alcohol screening and brief intervention in social services and workplace settings [49]. The review concluded that non–health care settings might result in additional implementation challenges in regard to the provider level, in this case the job consultants. Unlike those in medical settings that focus specifically on alcohol-related problems, social providers might not feel responsible for delivering alcohol-related interventions as part of their job description. Some of the same tendencies among health care professionals have been shown by other studies [48,61] and are also demonstrated in this paper.

In this study each municipality had its own way of organising, administering, and managing its work. We tried to embrace these differences while remaining true to the overall study design. However, there was a mixture of welfare recipients with different needs in all four municipalities. Job consultants highlighted that some welfare recipients had more-serious problems than alcohol, while other were young non-treatment-seeking welfare recipients who could not relate to the project. The identified heterogeneous nature of a non–health care setting and the complexity of certain welfare recipients’ needs potentially suggest that approaches will need to be tailored to different contexts [49] rather than standardised across all group [67]. This is probably less than realistic, if interventions simultaneously should be implemented into routine practice and under realistic conditions in Danish job centres. Unless extra resources are allocated

The project Alcohol and Employment is an attempt to obtain knowledge that can facilitate wider implementation of alcohol screening and brief intervention in social services. This paper outlines major barriers to the implementation of the project. As demonstrated above, our findings are consistent with barriers identified in other settings. These barriers may have prevented the optimal translation from occurring at the Danish job centres and may be the reason for the low enrolment number. Schulte’s (2014) systematic review [49] confirms problems with enrolment in non-health care settings, such as criminal justice, social services, and workplace settings. Future studies in similar pragmatic settings can take advantage of these findings when preparing interventions that are implemented into routine practice by job consultants or similar professionals.

### Limitations

One limitation of our analysis is that it draws only on the subset of data gathered to study the implementation of Alcohol and Employment. We recognise, however, that implementation is often a long and protracted process and factors that influence the adoption and implementation may change at different points in the process [26,27]. Second, the sampling strategy might have created bias in the results, if the participants were not representative for all the participating job consultants. It is our impression that the job consultants most critical of the project did not want to participate, and thus the analysis might create a more positive picture than reality. However, it is also our impression that participants referred to positions and actions of colleagues, thus covering a broader picture. Third, even though we aimed to include themes that we believed captured the most important factors, there may have been important organisational factors that we did not identify (e.g., political and administrative support and leadership, and organisational culture), which may be considered in future research. Fourth, the decision to participate in the project was taken at the management level at the job centres. This top-down approach may have failed to involve and establish the necessary ownership of the job consultants, which is a shortcoming of the project design.

## Conclusions

The Alcohol and Employment project was difficult to implement, which affected the recruitment of welfare recipients and job consultants for this innovative research initiative. This study has identified important factors at the individual level among the job consultants who threatened the implementation of Alcohol and Employment. Future studies in similar settings can take advantage of these findings when preparing interventions that are implemented by job consultants or similar professionals.
